# The Restorative Effects of Electron Mediators on the Formation of Electroactive Biofilms in *Geobacter sulfurreducens*

**DOI:** 10.3390/microorganisms14010214

**Published:** 2026-01-17

**Authors:** Zheng Zhuang, Yue Shi, Guiqin Yang, Li Zhuang

**Affiliations:** 1 School of Geography, Geomatics and Planning, Jiangsu Normal University, Xuzhou 221116, China; zhuangzheng@jsnu.edu.cn (Z.Z.); shiyue10320@163.com (Y.S.); 2 Guangdong Key Laboratory of Environmental Pollution and Health, School of Environment, Jinan University, Guangzhou 511443, China; gqyang@jnu.edu.cn

**Keywords:** *Geobacter sulfurreducens*, electroactive biofilm, bioelectrochemical systems, electron mediator, extracellular electron transfer

## Abstract

Electroactive biofilms (EABs) are essential for the performance of bioelectrochemical systems (BESs), but their formation in *Geobacter*, critically on conductive pili and exopolysaccharides, limits application under conditions where these components are deficient. Herein, we investigated the restorative effects of exogenous flavin mononucleotide (FMN) on EAB formation and extracellular electron transfer (EET) in two defective mutants of *Geobacter sulfurreducens*: the pili-deficient PCAΔ1496 and exopolysaccharides-deficient PCAΔ1501. Results show that FMN significantly promoted biofilm thickness in PCAΔ1496 (250%) and PCAΔ1501 (33%), while boosting maximum current outputs by 175-fold and 317.7%, respectively. Spectroscopic and electrochemical analyses revealed that FMN incorporates into biofilms, binds to outer membrane *c*-type cytochromes (*c*-Cyts), and enhances electron exchange capacity. Differential pulse voltammetry further confirmed that FMN did not exist independently in the biofilm but bound to outer membrane *c*-Cyts as a cofactor. Collectively, exogenous FMN plays dual roles (electron shuttle and cytochrome-bound cofactor) in defective *Geobacter* EABs, effectively restoring biofilm formation and enhancing EET efficiency. This study expands the understanding of the formation mechanism of *Geobacter* EABs and provides a novel strategy for optimizing BES performance.

## 1. Introduction

More than 99% of microorganisms on earth exist in the form of biofilm, which drives almost all biogeochemical processes [1]. Compared with planktonic microorganisms, biofilms have more complex structures, wider information exchange and more sophisticated regulatory mechanisms [2]. In bioelectrochemical systems (BESs), electroactive bacteria can aggregate on the electrode surface to form electroactive biofilms (EABs) with efficient electron transfer capabilities, which is hundreds of times higher than that of planktonic microorganisms [3]. EABs are the main driving force for energy generation by BESs and play a crucial role in their effective application [4]. During the last few decades, BESs have shown non-negligible potential in various applications such as power supply, wastewater treatment, noble metal reduction, and contaminated soil remediation [5,6]. However, the limited current density and energy efficiency of BESs remain a difficult challenge to achieve their commercial application [7].

The thickness of EABs is closely related to the energy output of BESs, and the formation of mature EABs is one of the prerequisites for improving the performance of BESs [8,9]. It is generally accepted that the conductive pili of electroactive bacteria are required for the formation of EABs. Conductive pili not only contribute to intercellular aggregation and structural support of biofilms, but also facilitate long-range extracellular electron transport in biofilms due to their elongated structure and high conductivity [10,11]. Recent studies have demonstrated that non-conductive exopolysaccharides of electroactive bacteria are indispensable in early biofilm formation. Moreover, exopolysaccharides enhance the electrochemical activity of biofilms by anchoring extracellular *c*-type cytochromes (*c*-Cyts) and maintaining cell viability [12,13]. Therefore, it is widely recognized that conductive pili and exopolysaccharides are indispensable biological factors for the formation of EABs.

Electron mediators (EMs), represented by flavin mononucleotide (FMN), are soluble organic substances that can participate in electron transfer between electroactive bacteria and electron acceptors by virtue of their inherent redox properties [14]. *Shewanella* and *Geobacter*, as the most representative electroactive bacteria in BESs, have attracted widespread attention from researchers regarding their interaction with EMs [15]. Studies on *Shewanella* have found that in addition to the well-known electron shuttle process, EMs can also induce the formation of thick EABs [16,17]. Multiple EMs, including FMN, riboflavin (RF), 9,10-anthraquinone-2,6-disulfonic acid (AQDS), 2-hydroxy-1,4-naphthoquinone (2-HNQ) and 9,10-anthraquinone-2-sulfonic acid (AQS), can effectively promote the generation of higher current density and thicker biofilms by *Shewanella* [17]. However, there is currently no direct evidence indicating that exogenous EMs can promote the formation of biofilms in *Geobacter*. Particularly, under conditions where a crucial biological factor (such as pili or exopolysaccharides) is absent, the functional role that EMs might assume remains unclear.

In this study, pili-deficient mutant and exopolysaccharides-deficient mutant of *G. sulfurreducens* were constructed separately, both of which were unable to form mature and high-performance EABs in BESs. By adding exogenous FMN to BESs, we explored the role of FMN in the formation of *Geobacter* EABs. Furthermore, combined with microbial electrochemical technology, we investigated the binding state of FMN with extracellular *c*-Cyts and its impact on extracellular electron transfer (EET). This work contributes to expanding the understanding of the classical formation mechanism of *Geobacter* EABs, providing a new perspective for better optimizing the performance of BESs.

## 2. Materials and Methods

### 2.1. Bacterial Strains

Bacterial strains used in this study are listed in Table S1. *G. sulfurreducens* strain PCA (DSM 12127) was received from German Collection of Microorganisms and Cell Cultures (DSMZ) (Braunschweig, Germany) and used to construct pili-deficient mutant and exopolysaccharides-deficient mutant. All *Geobacter* strains were cultured in NBAF medium at 30 °C, and the medium was anaerobic treated (80/20 N_2_-CO_2_) before use [18]. *Escherichia coli* strain DH5α (Tsingke Biological Technology, Beijing, China) was used for routine cloning and cultured in LB broth medium at 37 °C.

### 2.2. Mutants Construction

All primers and plasmids applied to construct the mutant are listed in Table S1. Exopolysaccharides-deficient mutant PCAΔ1501 was constructed following the method in our previous study [12]. For the construction of pili-deficient mutant PCAΔ1496, three fragments were prepared: the primer pairs 1496 upf/1496 upr and 1496 dnf/1496 dnr were used to amplify the sequences 500 bp upstream and downstream, respectively, of GSU1496, using strain PCA genomic DNA as a template, and the primer pair Kmf/Kmr was used to amplify the kanamycin resistance cassette from the plasmid pET-28a. The three fragments and linearize plasmid pUC19 were seamlessly connected to generate plasmid pUC19-1496Km using the In-Fusion HD Cloning Kit (Takara Biomedical Technology, Kusatsu, Japan). This plasmid was linearized with *BseYI* (New England Biolabs, Ipswich, MA, USA) and then electroporated into electrocompetent *G. sulfurreducens* strain PCA, generating the mutant PCAΔ1496. The mutant PCAΔ1496 was verified by PCR and sequencing.

### 2.3. BESs Construction and Biofilm Formation

Single-chamber BESs were constructed for current generation experiments and biofilm cultivation [19]. The reactors were monitored using a multichannel potentiostat (CHI1010C, CH Instruments, Shanghai, China) and operated under potentiostatic control, with a saturated calomel electrode (SCE, +0.2415 V vs. standard hydrogen electrode, SHE) serving as the reference electrode. Polished graphite plates (30 mm × 15 mm × 5 mm) were employed as both the working and counter electrodes, with the working electrode poised at +0.2 V (vs. SCE). The reactors were operated in batch mode, and the electrolyte was replaced with fresh medium once the current dropped below 10^−4^ A (defining one batch cycle). For the initial cycle, the electrolyte consisted of a freshwater medium containing acetate (15 mM) as the electron donor and fumarate (40 mM) as the electron acceptor. In subsequent cycles, the electrolyte contained only acetate (15 mM) as the electron donor. Each reactor was filled with 70 mL of electrolyte and maintained under strictly anaerobic conditions. To investigate the effect of FMN, an additional 50 μM FMN was introduced into the electrolyte. All BESs were operated at a constant temperature of 30 °C.

### 2.4. Biofilm Imaging

When the current of the BESs stabilized, the growth and thickness of the biofilm were examined using a confocal laser scanning microscope (CLSM, LSM 800, Carl Zeiss, Oberkochen, Germany). For sample preparation, the biofilms were stained with the LIVE/DEAD BacLight Bacterial Viability Kit (Thermo Fisher Scientific, Waltham, MA, USA) in the dark for 15 min, followed by gentle rinsing with PBS to remove excess dye. Multi-layer continuous scanning of the stained biofilms was performed using CLSM, yielding a set of consecutive z-series images. The z-stack interval was set to 0.5 μm, and each sample was scanned across 5 randomly selected non-overlapping regions (100 × 100 μm per region) to avoid sampling bias. Biofilm thickness was quantified using Zeiss ZEN 3.0 (Blue Edition) software, and thickness was calculated as the vertical distance between the top (highest fluorescent z-plane) and bottom (electrode surface reference) boundaries.

### 2.5. Electrochemical Measurements

The electrochemical properties of the anode biofilm under turnover (highest current point) and non-turnover conditions (lowest current point) were measured using a CHI660E electrochemical workstation (CH Instruments), following the previously reported method [20]. Differential pulse voltammetry (DPV) was performed with the following parameters: Init E = −0.8 V, Final E = 0.3 V, Ince E = 0.002 V, Amplitude = 0.05 V, Pulse Width = 0.25 s, Sampling Width = 0.02 s, Pulse Period = 0.5 s, Quiet Time = 0 s, Sensitivity = 1 × 10^−3^ A/V.

### 2.6. Extraction and Determination of Extracellular Polymeric Substances

The biofilm on the working electrode was scraped off and resuspended in 0.9% NaCl after the current of BESs stabilized. The same volume of 2% Na2 EDTA was added to the suspension and mixed evenly. The mixture was incubated at 4 °C for 3 h and then centrifuged at 5000× *g* at 4 °C for 20 min. All supernatants were filtered through a 0.22 μm membrane filter (polyethersulfone) to obtain extracellular polymeric substances (EPSs) solution with bacteria and impurities removed [12]. The electron donating capacity (EDC) and electron accepting capacity (EAC) of EPSs are measured to characterize the electron exchange capacity of EPSs, as previously reported in [21].

### 2.7. c-Cyts Analysis

The electronic absorption spectra of EPSs were acquired using a UV-Vis spectrophotometer (UV-2600, Shimadzu, Kyoto, Japan) in the wavelength range of 350–650 nm. For oxidation state characterization, control EPS samples (exposed to ambient air) were designated as oxidized EPS, while reduced EPS samples were prepared by treatment with sodium dithionite. The UV-Vis spectra of oxidized EPSs were measured directly, followed by spectroscopic analysis of the sodium dithionite-reduced EPS samples.

### 2.8. Statistical Analysis

Data were obtained from three independent biological replicates and are presented as mean ± standard deviation (SD). Statistical analyses were conducted using SPSS 19.0 software. One-way analysis of variance (one-way ANOVA) followed by the least significant difference (LSD) post hoc test were employed to determine statistically significant differences among groups. Differences with the control group were considered significant when *p* < 0.05, *p* < 0.01, and *p* < 0.001, with significance levels indicated by asterisks *, **, and ***, respectively.

## 3. Results

### 3.1. Exogenous FMN Promoted Biofilm Formation

**Figure 1:**
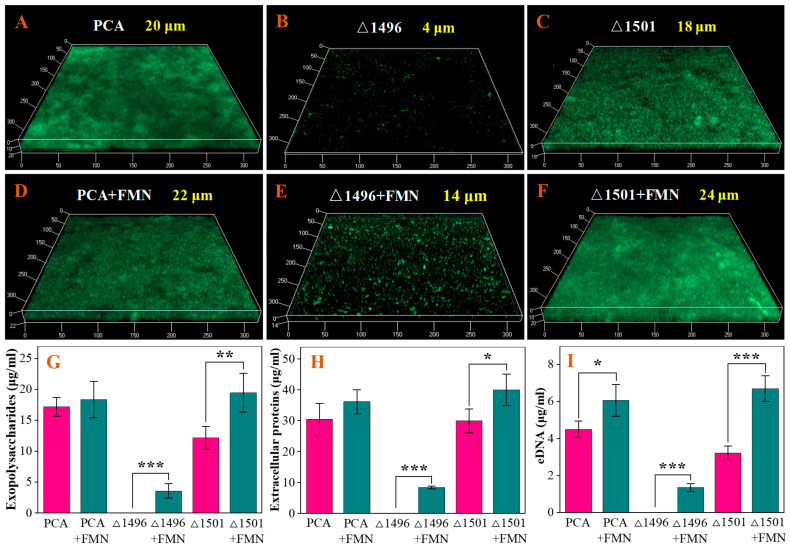
3D CLSM images showing the biofilm thickness of strains PCA (**A**,**D**), PCAΔ1496 (**B**,**E**), and PCAΔ1501 (**C**,**F**) in the absence/presence of exogenous FMN, and concentrations of extracellular polysaccharides (**G**), extracellular proteins (**H**), and eDNA (**I**) of all strains under the two conditions. Differences with the control group were considered significant when *p* < 0.05, *p* < 0.01, and *p* < 0.001, with significance levels indicated by asterisks *, **, and ***, respectively.

The thickness of biofilms cultured for four cycles was observed using CLSM. The pili-deficient mutant PCAΔ1496 and exopolysaccharides-deficient mutant PCAΔ1501 of *G. sulfurreducens* formed two distinct types of defective biofilms. Specifically, the pili-deficient mutant PCAΔ1496 could only develop a biofilm with a thickness of several micrometers after prolonged cultivation (Figure 1B). In contrast, the exopolysaccharides-deficient mutant PCAΔ1501 exhibited significantly restricted biofilm formation at the early cultivation stage (Figure S1B,D), but was capable of forming a relatively thick biofilm after prolonged cultivation (Figure 1C). The addition of exogenous FMN exhibited minimal impact on the biofilm thickness of the wild-type strain PCA (Figure 1A,D). Notably, exogenous FMN increased the thickness of PCAΔ1496 and PCAΔ1501 biofilms by 250% (Figure 1B,E) and 33% (Figure 1C,F), respectively. These phenomena indicate that exogenous FMN has a significant restorative effect on the growth of defective biofilms.

EPS are high-molecular-weight polymers secreted by microorganisms into the extracellular environment, primarily comprising polysaccharides, proteins, and nucleic acids [22]. Determination of the concentrations of extracellular polysaccharides, extracellular proteins, and extracellular DNA (eDNA) in EPSs from different types of biofilms showed that exogenous FMN increased the concentrations of each EPS component in the biofilms of PCA, PCAΔ1496, and PCAΔ1501 to varying degrees. Among them, the promoting effect on PCA biofilms was relatively weaker, while it was relatively stronger on PCAΔ1496 and PCAΔ1501 biofilms (Figure 1G–I), which was consistent with the results of biofilm growth and development (Figure 1A–F).

### 3.2. Exogenous FMN Increased EET Efficiency

**Figure 2:**
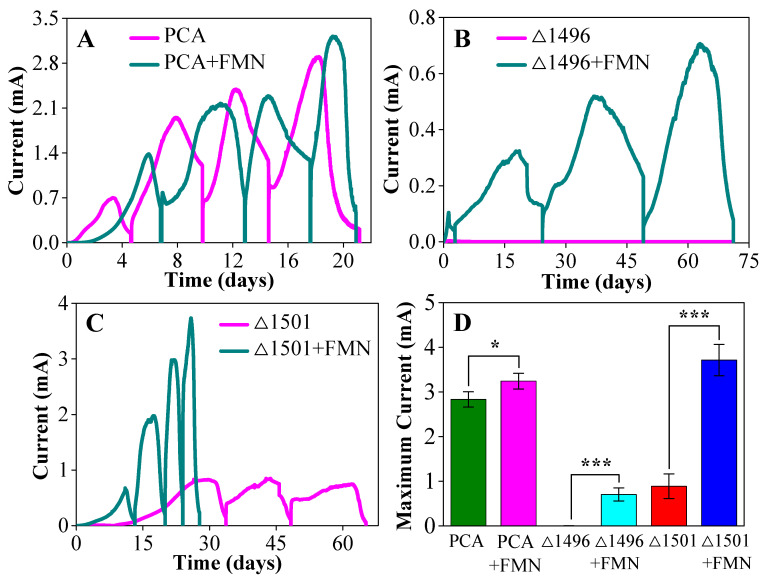
Real-time current curves of strains PCA (**A**), PCAΔ1496 (**B**), PCAΔ1501 (**C**), along with their maximum current (**D**). Differences with the control group were considered significant when *p* < 0.05, and *p* < 0.001, with significance levels indicated by asterisks *, and ***, respectively.

To clarify the effect of exogenous FMN on the electron transfer of the strains, we monitored their electricity production process and maximum current. As shown in Figure 2A–C, after four cycles of cultivation, exogenous FMN promoted the electricity production of *G. sulfurreducens* wild-type strain PCA, pili-deficient mutant PCAΔ1496, and exopolysaccharides-deficient mutant PCAΔ1501 to varying degrees. Exogenous FMN increased the maximum current of PCA by 15.5%, but compared with PCAΔ1496 and PCAΔ1501, the promoting effect of exogenous FMN on PCA electricity production is relatively weaker (Figure 2D). The promoting effect of exogenous FMN on the electricity production of PCAΔ1496 and PCAΔ1501 was highly significant. Specifically, PCAΔ1496 generated only a weak current (0.004 mA) in the first cycle and almost no current in the subsequent three cycles, while exogenous FMN enhanced its maximum current by 175-fold (Figure 2B,D). For PCAΔ1501, exogenous FMN not only increased the maximum current by 317.7% but also shortened its electricity production cycle by more than half (Figure 2C,D).

### 3.3. Exogenous FMN Enhanced Biofilm Electroactivity

**Figure 3:**
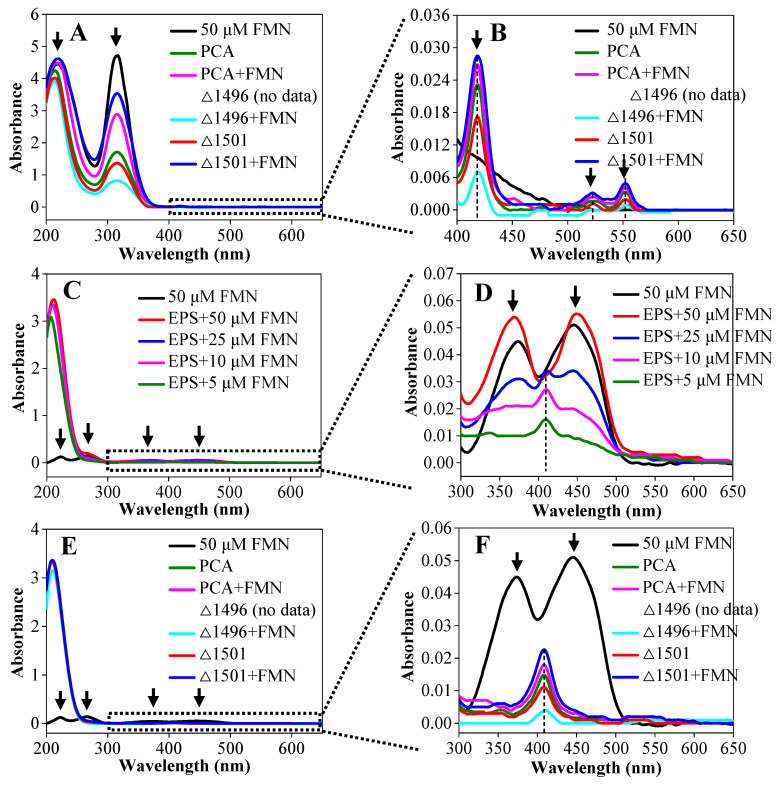
UV-Vis spectra of reduced EPS (**A**), oxidized EPS (**C**) and oxidized EPS (**E**) under different treatments, with magnified insets of (**A**), (**C**) and (**E**) corresponding to (**B**), (**D**) and (**F**), respectively. The arrows in the figures represent the UV-Vis characteristic peaks of *c*-Cyts or FMN, and the vertical dashed lines mark the absorbance value at which the characteristic peak is located.

*c*-Cyts in EPS, present in oxidized and reduced forms, can be quantified by measuring their relative levels using UV-Vis spectroscopy. Figure 3A,B presents the UV-Vis spectra of the reduced EPS. The control sample (50 μM FMN) exhibited two distinct characteristic peaks at 218 nm and 318 nm. The EPS sample also showed pronounced peaks at identical wavelengths (Figure 3A), indicating that the exogenous FMN was incorporated into the biofilm. Furthermore, the EPS sample displayed three characteristic peaks at 419 nm, 522 nm, and 552 nm (Figure 3B), which were identified as signatures of reduced *c*-Cyts [23]. Since the absorbance of characteristic peaks for both *c*-Cyts and FMN is proportional to their concentration, the relative abundance of reduced *c*-Cyts and FMN across different treatments followed the order: PCAΔ1501+ FMN > PCA + FMN > PCA > PCAΔ1501 > PCAΔ1496 + FMN.

To validate the relationship between the characteristic UV-Vis peaks of *c*-Cyts and FMN in oxidized EPS samples, spectral scans were performed on FMN solution and EPS samples supplemented with varying concentrations of FMN. As shown in Figure 3C, the control sample (50 μM FMN) exhibited four distinct characteristic peaks at 223 nm, 266 nm, 374 nm, and 445 nm, which correspond to oxidized FMN [24]. Figure 3D illustrates that at an FMN concentration of 50 μM, the characteristic peaks of FMN masked those of *c*-Cyts. When the FMN concentration was 25 μM, the characteristic peaks of both FMN and *c*-Cyts were simultaneously present. With the FMN concentration further reduced to 5 μM, the characteristic peaks of *c*-Cyts became dominant and masked those of FMN. In summary, the characteristic peaks of *c*-Cyts and FMN can mutually mask each other depending on the concentration of FMN present. The UV-Vis spectra of oxidized EPSs are presented in Figure 3E. The control sample (50 μM FMN) exhibited four distinct characteristic peaks of oxidized FMN at 223 nm, 266 nm, 374 nm, and 445 nm. In contrast to the reduced EPS spectra (Figure 3A), the EPS sample lacked these oxidized FMN peaks (Figure 3E). Combined with the data from Figure 3C,D, this absence is attributed to the masking of FMN characteristic peaks by those of *c*-Cyts. Based on comparative spectral analysis with FMN standards, the concentration of FMN accumulated in EPS was inferred to be below 5 μM. As shown in Figure 3F, the relative abundance of oxidized *c*-Cyts and FMN across different treatments followed the order: PCAΔ1501 + FMN > PCA + FMN > PCA > PCAΔ1501 > PCAΔ1496 + FMN. This order is identical to that observed for the reduced forms and is consistent with the trend in biofilm thickness (Figure 1A–F).

The sum of EAC and EDC values is defined as the electron exchange capacity, representing the total number of redox-active mediators in the sample [25]. The addition of exogenous FMN increased the EAC and EDC of EPS in PCA, PCAΔ1496, and PCAΔ1501 biofilms to varying degrees, with a more substantial effect observed in the two mutant strains compared to the wild-type PCA (Figure S2A,B). These elevated EAC and EDC levels correspond to a higher content of *c*-Cyts and FMN, aligning with the UV-Vis spectral data (Figure 3A–F). Furthermore, significant positive correlations (*p* < 0.001) were found between the maximum current and both EAC and EDC (Figure S2C,D).

### 3.4. Exogenous FMN Acted as Cytochrome-Bound Cofactors

**Figure 4:**
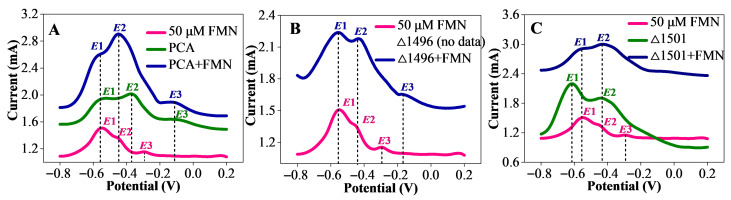
DPV results of biofilms formed by strains PCA (**A**), PCAΔ1496 (**B**), and PCAΔ1501 (**C**) in the absence/presence of FMN, with all DPV scans performed under non-turnover conditions.

DPV is considered an electrochemical method with high selectivity and sensitivity, commonly used to study the redox potential of redox active substances in EABs [26]. DPV scans were performed on different types of biofilms and 50 μM FMN solution. The results showed that the FMN solution exhibited three redox potentials (E1, E2, and E3). For the PCA biofilm, its E1 position was identical to that of the FMN solution; upon FMN addition, the E2 position of the PCA biofilm shifted leftward and coincided with that of FMN (Figure 4A). The addition of FMN led to the coincidence of the E2 position between the PCAΔ1496 biofilm and the FMN solution (Figure 4B). The E2 position of the PCAΔ1501 biofilm was the same as that of the FMN solution; after FMN supplementation, the E1 position of the PCAΔ1501 biofilm shifted rightward and overlapped with that of the FMN solution (Figure 4C). Shifts in redox potentials are often caused by the binding of riboflavin to cytochromes [27]. Thus, the above results indicate that exogenous FMN entering the biofilm does not exist independently but binds to the outer membrane *c*-Cyts of bacteria.

## 4. Discussion

The negligible impact of exogenous FMN on the biofilm thickness of wild-type PCA (Figure 1A,D) aligns with the understanding that *Geobacter* species primarily rely on conductive pili and cytochromes for energy metabolism after forming mature biofilm, rather than on electron shuttles [16]. In contrast, exogenous FMN significantly promoted the growth of biofilms in the two mutants. This significant promotion was evident throughout the entire stage of biofilm formation in pili-deficient mutant and in the early stage of biofilm formation in exopolysaccharides-deficient mutant, indicating that FMN compensates for structural deficiencies in biofilm formation (Figure S1 and Figure 1B,C,E,F). This effective restoration of biofilm growth in the mutant strains may be attributed to the FMN in the suspension being rapidly reduced by electroactive microorganisms and subsequently oxidized by the electrode, creating a microenvironment with abundant oxidized electron shuttles that favors biofilm formation [17]. As a major structural component of biofilms, EPS content generally exhibits a positive correlation with biofilm thickness under normal conditions. The increase in EPS content across all strains upon FMN addition (Figure 1G–I) supports the role of EPS in enhancing biofilm electroactivity. The increase in EPS content facilitates the enhancement of biofilm electroactivity, as EPS not only protects microorganisms from environmental stress but also acts as mediators or conductors for electron transfer to maintain EET function [28].

The performance of BESs largely depends on the electroactive biofilms grown on the electrode surface, while the efficiency of EET from electroactive microorganisms to the electrode determines the electrochemical activity of the biofilms [29]. When the EET pathways of the biofilms are impaired, the addition of exogenous redox-active substances is generally regarded as an effective alternative strategy [30]. The differential promotion of electricity production between the wild-type and mutant strains reflects the different roles of FMN in EET (Figure 2A–D). For mature wild-type biofilms, the enhancement of their electricity production capacity by exogenous FMN is limited. Previous studies have demonstrated the regulatory genes involved in flavin biosynthesis and secretion in *G. sulfurreducens*, and *G. uraniireducens* has been found to secrete large amounts of flavins [31,32]. However, endogenously secreted flavins obviously do not dominate the electron transfer process of *Geobacter* [16]. Our experimental results also confirm that in mature *Geobacter* biofilms, closely stacked electroactive microorganisms mainly transfer electrons to the electrode via conductive pili and *c*-Cyts, with a minor contribution from electron shuttles. However, the dramatic enhancement of EET efficiency in mutants is due to FMN compensating for the defective electron transfer pathways. The electron transfer pathways of *Geobacter* biofilms vary with developmental stages: indirect electron transfer relies on flavin molecules in the early stage [33], while direct electron transfer occurs via *c*-Cyts and conductive pili in the late. When biofilm formation is restricted (e.g., lack of pili or exopolysaccharides), FMN acts as a substitute in the electron transfer process.

The UV-Vis spectral results confirm that exogenous FMN is incorporated into the biofilm and coexists with *c*-Cyts (Figure 3A–F). The mutual masking of characteristic peaks between *c*-Cyts and FMN depending on FMN concentration indicates an interaction between the two substances. The absence of oxidized FMN peaks in EPS samples suggests that the accumulated FMN concentration in EPS is below 5 μM, and the characteristic peaks of FMN are masked by those of *c*-Cyts. As an important component of EPS, *c*-Cyts with redox properties make significant contributions to the EET process of electroactive microorganisms, and their abundance largely determines the electroactivity of biofilms [34]. The elevated EAC and EDC levels in biofilms added with FMN is consistent with the increased contents of redox-active mediators, which positively correlate with maximum current output (Figure S2A–D), indicating that the enrichment of redox mediators in the biofilm promotes electron transfer efficiency and boosts electroactivity of the biofilms [35]. The shift and coincidence of redox potentials in DPV results verify that FMN does not exist independently in the biofilm but binds to outer membrane *c*-Cyts as a cofactor (Figure 4A–C). Okamoto et al. first discovered that flavins secreted by *S. oneidensis* can act as binding cofactors for outer membrane *c*-Cyts, and the interaction between flavins and outer membrane *c*-Cyts is conducive to regulating the EET process and intracellular metabolic activities [27]. Shortly thereafter, Okamoto et al. further confirmed that *G. sulfurreducens* can also secrete and utilize riboflavin as a binding cofactor for outer membrane *c*-Cyts to participate in the EET process [36]. In summary, under conditions of restricted biofilm formation, exogenous FMN not only can act as an electron shuttle to transfer electrons in the initial stage of biofilm formation but also can bind to *c*-Cyts during biofilm development to assist their electron transfer function.

## 5. Conclusions

In this study, we systematically investigated the restorative role of exogenous FMN on the formation of EABs in *G. sulfurreducens* mutants deficient in conductive pili (PCAΔ1496) or exopolysaccharides (PCAΔ1501). The results demonstrate that FMN not only compensates for structural deficiencies in biofilm formation but also significantly enhances the electron transfer capacity of the defective biofilms. Specifically, FMN increased biofilm thickness in PCAΔ1496 and PCAΔ1501 by 250% and 33%, respectively, and boosted their maximum current outputs by 175-fold and 317.7%, highlighting its potent restorative effect under conditions where key biofilm components are absent.

Mechanistic insights revealed that FMN operates through dual pathways, as a soluble electron shuttle facilitating intermediate electron transfer during early biofilm development and as a bound cofactor associated with outer membrane *c*-Cyts, thereby enhancing their redox activity within the biofilm matrix. UV-Vis and electrochemical analyses confirmed the incorporation and redox interaction of FMN with *c*-Cyts, while the significant positive correlations between electron exchange capacity (EAC/EDC) and current output underscore the role of FMN in augmenting the electroactivity of biofilms.

This work provides direct evidence that exogenous EMs can functionally restore and enhance biofilm formation and EET efficiency in *Geobacter* under deficiency conditions, broadening the conventional understanding of EAB development which has largely emphasized conductive pili and exopolysaccharides. Our findings propose a novel bioaugmentation strategy, namely the use of small molecular mediators such as FMN, to improve the performance and stability of BESs, particularly under suboptimal biofilm-forming conditions. Future studies may explore the synergistic effects of multiple mediators or engineered mediator-secreting strains to further advance the practical application of BESs in energy and environmental biotechnology.

## Data Availability

The original contributions presented in this study are included in the article/Supplementary Materials. Further inquiries can be directed to the corresponding author.

## Supplementary Materials

The following supporting information can be downloaded at: https://www.mdpi.com/article/10.3390/microorganisms14010214/s1. Table S1. Strains, plasmids, and primers used in this study; Figure S1. 3D CLSM images showing the biofilm thickness of strains PCAΔ1496 (A), PCAΔ1501 (B), PCAΔ1496 + FMN (C) and PCAΔ1501 + FMN (D) in the early stage of development. Figure S2. The electron exchange capacity of EPS from different types of biofilms is characterized by EAC (A) and EDC (B), with correlation analysis of EAC (C) and EDC (D) with the maximum current.
