# Supplementary material for: The Restorative Effects of Electron Mediators on the Formation of Electroactive Biofilms in Geobacter sulfurreducens
Source: Microorganisms. 2026 Jan 17;14(1):214. doi: 10.3390/microorganisms14010214 (PMC12843962; doi:10.3390/microorganisms14010214)
Supplement: Supplementary file 1 [file microorganisms-14-00214-s001.zip › microorganisms-4086846-supplementary.pdf]

Supplementary Materials

Article

The Restorative Effects of Electron Mediators on the Formation of Electroactive Biofilms in *Geobacter sulfurreducens*

Zheng Zhuang [1], Yue Shi [1], Guiqin Yang [2], Li Zhuang [2,*]

[1] School of Geography, Geomatics and Planning, Jiangsu Normal University, Xuzhou 221116, China; zhuangzheng@jsnu.edu.cn (Z.Z.); shiyue10320@163.com (Y.S.)
[2] Guangdong Key Laboratory of Environmental Pollution and Health, School of Environment, Jinan University, Guangzhou 511443, China; gqyang@jnu.edu.cn

* Correspondence: zhuangli@jnu.edu.cn

Table S1. Strains, plasmids, and primers used in this study

Strain, plasmid or primer	Relevant characteristics or sequence (5’ to 3’)	Source or usage
Strains		
E.coli		
DH5α	*F-, φ 80dlacZ ΔM15, Δ(lacZYA -argF) U169, deoR, recA1, endA1, hsdR17 (rK-, mK), phoA, supE44, λ-, thi -1, gyrA96, relA1*	Tsingke
G. sulfurreducens		
PCA	Wild-type strain	Lab stock
PCA△1496	△*GSU1496::Km*^{r}	This work
PCA△1501	△*GSU1501::Gm*^{r}	This work
Plasmids		
pET-28a	Kanamycin resistance gene, *Km*^{r}	Lab stock
pUC19	In-Fusion Cloning vector, *Amp*^{r}	Lab stock
pUC19-1496Km	pUC19 carrying 500bp upstream and downstream of GSU1496 and kanamycin resistance cassette, *Km*^{r}	This work
Primers		
kmf	GACGCTCAGTGGAACGAA	PCR amplification of kanamycin resistance gene
kmr	GCGGAACCCCTATTTGTT	
1496upf	cggtacccggggatcCCTACAGCGACCGCATCAT	PCR amplification of GSU1496 upstream
1496upr	gttccactgagcgtcGCTGCTTGTGTTGGGGTAT	
1496dnf	aaataggggttccgcCACCCTTATCGAGCTGCTG	PCR amplification of GSU1496 downstream
1496dnr	cgactctagaggatcTGACTACTGCGACTTCCAC	
M13f	TGTAAAACGACGGCCAGT	Plasmids verification
M13r	CAGGAAACAGCTATGACC	
ver1496f	TGGAATACGGGATGAAGT	Mutant verification
ver1496r	AAGGCGTTTGCCGATGCT	

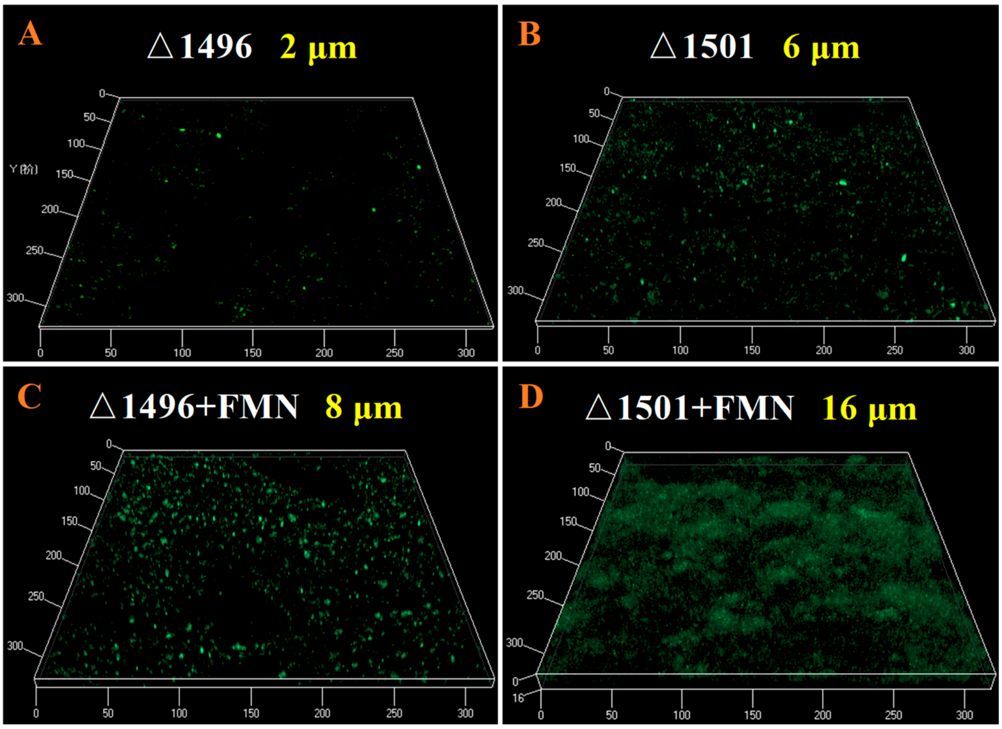

Figure S1. 3D CLSM images showing the biofilm thickness of strains PCA△1496 (A), PCA△1501 (B), PCA△1496 + FMN (C) and PCA△1501 + FMN (D) in the early stage of development.

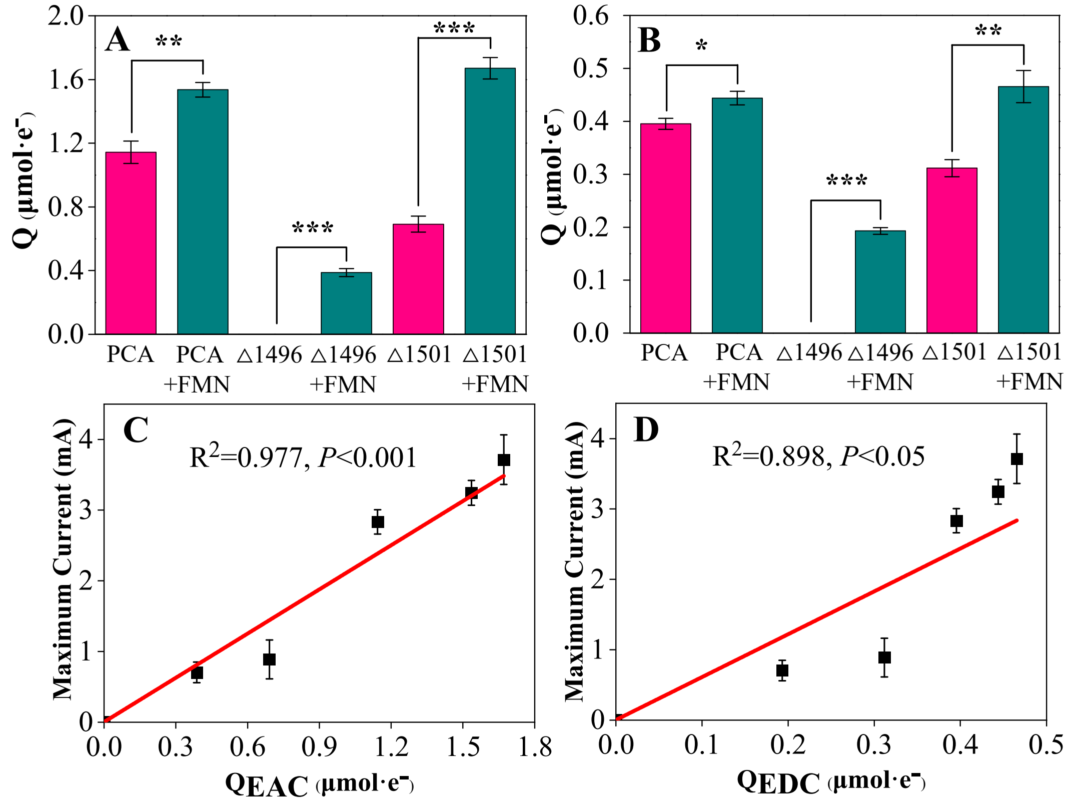

Figure S2. The electron exchange capacity of EPS from different types of biofilms is characterized by EAC (A) and EDC (B), with correlation analysis of EAC (C) and EDC (D) with the maximum current.
